# Wearable neurotechnology systems for upper extremity rehabilitation in children with cerebral palsy: a scoping review

**DOI:** 10.3389/fneur.2025.1663596

**Published:** 2025-11-05

**Authors:** Sara Jo Burchfield, Angela Shierk, Cindy Truong, Regan Blankenship

**Affiliations:** 1 Department of Occupational Therapy, The University of Texas Medical Branch, Galveston, TX, United States; 2 Department of Clinical Research, Scottish Rite for Children, Dallas, TX, United States; 3 Department of Occupational Therapy, Medical University of South Carolina, Charleston, SC, United States

**Keywords:** cerebral palsy, wearable neurotechnology, neurorehabilitation, pediatrics, upper extremity rehabilitation, occupational therapy

## Abstract

**Background:**

Children with cerebral palsy often experience persistent upper extremity impairments that impact independence and participation in daily activities. Wearable neurotechnology devices offer a promising, non-invasive approach to enhance motor control, promote neuroplasticity, and extend neurorehabilitation beyond clinical settings. However, the development and application of such devices in pediatric populations remains poorly defined. This scoping review aimed to map the existing literature on wearable neurotechnology systems used for upper extremity rehabilitation in children with cerebral palsy and identify knowledge gaps to guide future research and clinical translation in pediatric neurorehabilitation.

**Methods:**

This review followed the JBI Scoping Review Methodology and PRISMA-ScR guidelines. Four electronic database sources, MEDLINE, Scopus, CINAHL, and PsycINFO, were systematically searched to identify studies on wearable neurotechnology devices for upper extremity rehabilitation in children with cerebral palsy. Included studies consisted of journal articles published from January 2005 to June 2025, with full texts available in English and relevant gray literature sources. Data were extracted on neurotechnology characteristics, regulatory status, intervention protocols, and outcome measures.

**Results:**

From the 2,892 articles screened, 21 met the eligibility criteria. Most devices were in early developmental stages, with only five receiving regulatory approval. Studies examined various systems, including electromyography-triggered stimulation, virtual reality, and robot-assisted devices with haptic or electrical stimulation, and wearable garments embedded with electrical or vibrotactile stimulators. Intervention protocols varied widely across studies in terms of treatment intensity, wear schedules, and co-interventions. Feasibility was generally positive across studies, with high adherence rates and minimal adverse events reported. Many studies reported improvements in motor outcomes, including enhanced grip strength, hand use, range of motion, grasp and release ability, and muscular recruitment.

**Conclusions:**

Wearable neurotechnology shows potential to augment upper extremity rehabilitation in children with cerebral palsy, particularly through systems that support task-specific, feedback-driven practice. However, translation to clinical practice is limited by heterogeneity in device design, lack of standardized protocols, and limited high-quality evidence. Future research should prioritize standardization, clinician-centered implementation studies, and long-term outcomes to support integration into pediatric care.

**Systematic review registration:**

https://osf.io/5qxpe.

## 1 Introduction

Cerebral palsy (CP) is the most common movement disorder among the pediatric population (1). Prevalence estimates vary, with global population-based studies reporting rates between 1 and 4 per 1,000 live births (1). A meta-analysis from the Global CP Prevalence Group provides a more refined estimate of ~1.6 per 1,000 live births in high-income settings, with higher rates of up to ~3.4 per 1,000 reported in some low- and middle-income regions (2). CP arises from congenital or acquired neurological insults during fetal or early infant brain development, resulting in a group of non-progressive neurological disorders characterized by impaired motor and postural control (3). Clinical presentation varies depending on the size and location of the brain lesion and may include neuromuscular deficits such as weakness, limited range of motion, spasticity, and the development of atypical fine and gross motor patterns (4). These motor impairments present challenges with reaching, grasping, bimanual coordination, and manipulation skills, thereby restricting the functional use of the upper extremities for participation in play and self-help activities (5).

Rehabilitation strategies for children with CP aim to improve motor control and functional independence, although many individuals experience lifelong impairments (6). Therapeutic interventions typically include task-oriented approaches such as Constraint-Induced Movement Therapy (CIMT) and Hand-Arm Bimanual Intensive Therapy (HABIT), both of which are designed to leverage principles of neuroplasticity, the brain's capacity to reorganize in response to experience, learning, or injury (7). These approaches have demonstrated efficacy in promoting motor recovery in children with CP; however, they rely on residual voluntary motor function and may be less suitable for children with more severe motor impairments (7).

Recent advancements in neuromodulation research have introduced new avenues for enhancing neuroplasticity through targeted stimulation of neural circuits (8). Neuromodulation refers to the process of altering neural activity via electrical, mechanical, or sensory input to influence brain function and behavior (8, 9). Parallel to these developments, the field of neurotechnology has expanded to encompass a range of devices that offer new strategies to address the persistent challenges of restoring motor functions following neurological injury (8, 9). In this context, neurotechnology refers to the use of technological systems that interact with the nervous system to restore, enhance, or modulate neural function (9). Neurotechnology can be broadly categorized into invasive systems, which involve surgical implantation (e.g., brain-computer interfaces), and non-invasive systems, which operate externally without surgical implantation or direct penetration of the skin (8–10). Non-invasive neurotechnology systems are of particular interest in pediatric populations due to their reduced risk and ease of use (9).

Wearable neurotechnology represents a subcategory of non-invasive systems characterized by their portability and ability to integrate into real-life contexts to support motor rehabilitation (11, 12). These devices often involve wearable garments embedded with surface electrodes and sensors that detect movement intention and deliver neuromodulatory inputs such as functional electrical stimulation (FES), neuromuscular electrical stimulation (NMES), or vibrotactile feedback (11–13). These neurotechnologies are frequently incorporated into electromechanical, robotic, and virtual reality-based systems that facilitate intensive, repetitive, and goal-directed training designed to drive motor learning (9). Their accessibility encourages at-home use, increasing rehabilitation opportunities through more frequent practice, and improving carryover into everyday activities (11).

Collectively, wearable neurotechnology devices converge on the goal of enhancing neurorehabilitation, defined as a multidisciplinary process aimed at restoring function and improving quality of life following neurological injury or disease (7). Neurorehabilitation often integrates therapeutic interventions with emerging technologies to promote adaptive neuroplastic changes and functional recovery (9, 10). For example, innovations such as closed-loop systems, which adjust stimulation parameters in real time based on physiological feedback, exemplify the potential for personalized, responsive treatment paradigms (12, 14).

While previous studies have shown wearable devices to improve upper extremity performance in adults with upper motor neuron injuries, there is limited synthesized research on the types of wearable neurotechnology available for children with CP and their effects on upper extremity outcomes (13, 15, 16). Furthermore, pediatric studies often lack clear and consolidated information regarding device specifications, training models, and outcomes relevant to clinical practice (10). These gaps complicate decision-making for clinicians seeking to adopt innovative approaches to neurorehabilitation in children with CP. Emerging wearable neurotechnology systems that facilitate movement with electrical stimulation, electromyography (EMG) biofeedback, or haptic feedback hold the potential to reshape pediatric neurorehabilitation and enhance volitional motor control. These devices could significantly impact the quality of life in children with CP by facilitating increased movement and use of the upper extremities, leading to increased independence and participation in meaningful occupations (17).

This scoping review aims to (i) map existing evidence on wearable, non-invasive neurotechnology devices used to improve upper extremity function in children with CP; (ii) define wearable, non-invasive neurotechnology in the context of motor rehabilitation for children with CP; and (iii) identify gaps to guide future research and clinical translation in pediatric neurorehabilitation.

## 2 Methods

### 2.1 Study design

This review followed the Joanna Briggs Institute (JBI) Scoping Review Methodology and the Preferred Reporting Items for Systematic Reviews and Meta-Analyses Extension for Scoping Reviews (PRISMA-ScR) guidelines (18, 19). A protocol was prospectively registered with the Open Science Framework (https://osf.io/5qxpe).

### 2.2 Search strategy

A comprehensive search strategy was developed in collaboration with a medical librarian and applied across four databases: MEDLINE, Scopus, CINAHL, and PsycINFO, with formatting tailored to each database. These databases were selected to capture studies related to neurological disorders, pediatrics, rehabilitation, and technology. Search terms included combinations of keywords such as “cerebral palsy,” “neurotechnology,” “rehabilitation,” and “children.” The full list of search terms and strategies is provided in Supplementary material 1. To ensure an exhaustive search, an additional literature search was conducted by hand searching in Google Scholar and PubMed, using the same search terms. Reference lists of included studies and relevant systematic reviews were also screened for potentially eligible studies. To supplement peer-reviewed literature and address potential publication bias, gray literature sources were also searched by reviewing conference proceedings, organizational reports, and market research related to neurotechnology development and commercially available devices. This involved targeted searches of neurotechnology devices, companies, researchers, and manufacturers identified in the included studies and related systematic reviews. The literature search was completed on May 8, 2025.

### 2.3 Study selection

**Figure 1:**
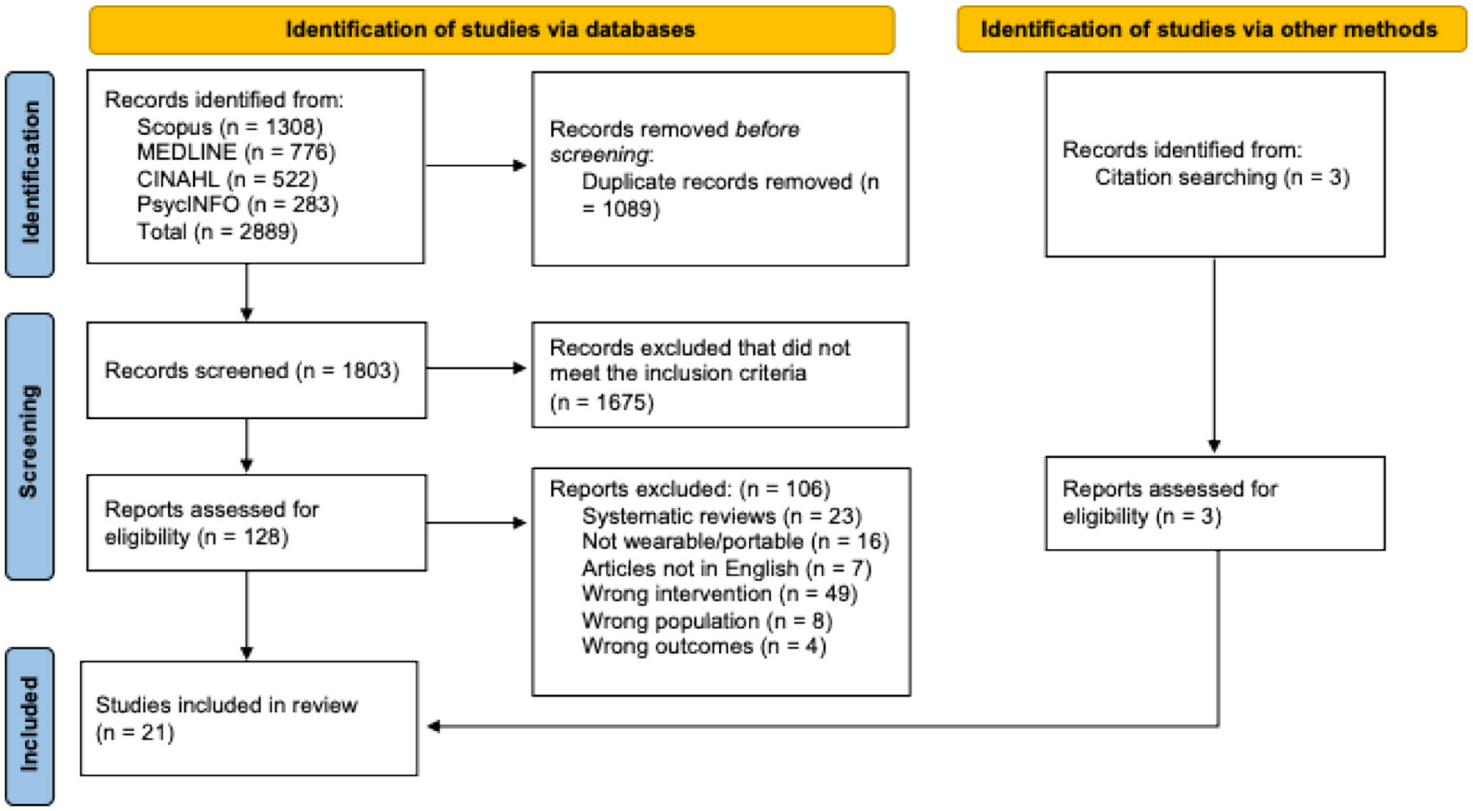
PRISMA flow diagram representing the study selection phases.

Articles were compiled into Covidence Systematic Review Software (Veritas Health Innovation, Melbourne, Australia), where duplicates were removed. Articles were then screened in two phases and checked for reliability by the research team. Two independent reviewers screened article titles and abstracts for eligibility and assessed the remaining articles' full texts for eligibility criteria detailed in Table 1. A third reviewer resolved any disputes through blind voting and a consensus discussion. Reasons for exclusion were documented during the full text phase and included in the PRISMA flow diagram in Figure 1 (20).

**Table 1:** Key eligibility criteria for article screening and study selection.

**Research elements**	**Eligibility criteria**
Participants	Inclusion: Children and adolescents aged 21 years or younger with a diagnosis of cerebral palsy or prenatal or neonatal stroke who demonstrate motor impairments in one or both upper extremities. Studies containing a variety of diagnoses among pediatric populations were only included if at least one participant had a diagnosis of cerebral palsy.
	Exclusion: Participants with profound comorbidities such as severe intellectual disability, epilepsy, or significant sensory impairments were excluded due to possible contraindications for neurotechnology use for these populations.
Intervention	Inclusion: Wearable, non-invasive neurotechnology devices with an active bioelectric or neurostimulation component intended to improve upper extremity function. Neurotechnology systems combined with other upper extremity therapeutic methods, such as virtual reality, bimanual, and robotic-assisted therapy.
	Exclusion: Passive robotic devices, orthotics, or exoskeletons that support or monitor upper extremity function were excluded to narrow the focus on active neurorehabilitative techniques. Invasive neurotechnology techniques that require surgical procedures (e.g., implanted devices, brain-computer interfaces, or deep brain stimulation).
Context	Any setting (e.g., hospitals, homes, clinics, schools) and all countries were considered for all studies with full texts available in English.
Outcomes	Inclusion: Usability, feasibility, upper extremity functional measures (e.g., fine motor, strength, range of motion, bimanual function), and device characteristics (e.g., control unit, availability).
	Exclusion: Outcomes related to the use of neurotechnology to monitor assessment data or detect motor function, and not as a means to enhance motor function.
Evidence sources	Inclusion: Peer-reviewed articles (2005–2025), feasibility studies, pilot studies, randomized controlled trials, case reports/series, and gray literature such as conference proceedings, organizational reports, and market research.
	Exclusion: Systematic/scoping reviews were excluded, but their references were scanned for eligible studies. Conference abstracts, dissertations, and thesis papers were excluded.

Included studies consisted of peer-reviewed journal articles published from January 2005 to May 2025, with full text available in English. Considering the limited literature available, multiple study designs were included for synthesis, such as randomized controlled trials, controlled trials, longitudinal studies, case series, and case studies. Feasibility studies were included if they provided relevant information on the safety and usability of these devices for children with CP. Additionally, systematic and scoping reviews were excluded; however, relevant reviews were scanned for references that fit within our inclusion criteria.

The specific inclusion criteria for neurotechnology devices involved: (i) Wearable non-invasive neurotechnology devices for upper extremity rehabilitation, (ii) Devices that include electrical stimulation, haptic, or vibro-tactile biofeedback components applied to the skin or muscle bellies to enhance motor activation, and/or (iii) neurotechnology devices combined with other upper extremity therapeutic methods, such as virtual reality, bimanual therapy, and robotic-assisted therapy. Exclusion criteria included: (i) studies that only investigate passive robotic devices, orthotics, or exoskeletons that support the upper extremity, (ii) Invasive neurotechnology techniques that require surgical procedures, such as implanted devices, brain-computer interfaces, or deep brain stimulation, (iii) neurotechnologies that do not have a wearable component but are used to enhance upper extremity function, such as transcranial magnetic stimulation, peripheral magnetic stimulation, transcranial direct current stimulation, or other non-invasive brain stimulation techniques, (iv) Studies that only utilize wearable technology components to monitor assessment data and not as a means to facilitate upper extremity outcomes.

Although this search strategy was intentionally broad, the number of studies meeting the inclusion criteria remained limited. This highlights the early stage of research in this area and underscores the need for further investigation into wearable neurotechnology for upper extremity rehabilitation in children with CP.

### 2.4 Data extraction

Two independent reviewers extracted data from the eligible articles and organized relevant information into a detailed charting system that aligns with the research questions. Extracted data is presented in a comprehensive table that includes information about the author(s), year of publication, study design, population demographics, sample size, setting, interventions and dosage, control conditions, and any additional therapeutic techniques provided to participants. Outcome measures related to feasibility and upper extremity rehabilitation were also reported. Specific details about the types of neurotechnology systems used, how they are controlled, whether the devices are FDA approved, the price range and availability of the technology, and whether the study includes company-sponsored research were also extracted. Study protocols and gray literature that include data from websites and organizations that develop neurotechnology devices and market research regarding commercially available neurotechnology devices were used to locate supplementary information. The tertiary reviewer resolved disputes involving data extraction methods among reviewers through discussion.

### 2.5 Data synthesis

The results of data extraction were synthesized using descriptive analysis. The data were first organized into a comparative chart to facilitate cross-study evaluation of key variables and outcomes. A narrative synthesis was developed based on descriptive and thematic patterns related to neurotechnology characteristics, availability, upper extremity outcomes, and feasibility. The synthesis was conducted collaboratively among reviewers to identify strengths, limitations, and gaps in the use of wearable neurotechnology for upper extremity rehabilitation for children with CP.

## 3 Results

### 3.1 Literature search results

A total of 2,889 articles were identified across the databases searched, with an additional 3 articles located through citation searching. After removing 1,089 duplicates, 1,803 articles remained for title and abstract screening. Of these, 1,675 were excluded for not meeting the eligibility criteria. The remaining 131 articles underwent full-text review, resulting in 21 studies that met the inclusion criteria and were included in the synthesis (Figure 1).

Many studies were excluded during the initial screening phase due to the invasive nature of the neurotechnology designs or the use of electrical stimulation without a wearable component. Additional articles investigating brain stimulation methods or pharmaceutical interventions for spasticity reduction were excluded because they did not meet the eligibility criteria of this review. During full-text review, several studies were also excluded for lacking a bioelectric or feedback component within the technology system, such as those using handheld gaming controllers or virtual reality platforms alone. These exclusion criteria substantially narrowed the evidence base, contributing to the final inclusion of only 21 studies and highlighting the limited scope of current research in this area.

The included studies were published between 2009 and 2025 (Supplementary material 2). Study designs included three randomized controlled trials (21–23), one pre-test-post-test study (24), one cross-sectional study (25), three feasibility studies (26–28), four pilot studies (29–32), one longitudinal retrospective cohort study (33), four case series (34–37), and four case reports (38–41). Geographically, studies were conducted in the United States (*n* = 7), Switzerland (*n* = 4), Italy (*n* = 4), Japan (*n* = 2), and one each in Egypt, Taiwan, Romania, and Canada. Settings included rehabilitation centers, hospitals, outpatient clinics, and home or community environments.

### 3.2 Population demographics

Across the 21 included studies, a total of 293 participants were reported. This sample comprised 213 children with CP (ages 4–19) and 13 healthy controls. One study included both children and adults with CP (ages 9–38) (33), while another enrolled adults as healthy controls (26). In addition, eight studies included participants with other diagnoses, which contributed to the overall sample size (22, 24–27, 30, 33, 36). Gender was reported for most participants, with 169 males (59.3%) and 116 females (40.7%). Thirteen studies reported CP subtypes, including hemiplegic CP (*n* = 64), spastic CP (*n* = 95), dyskinetic CP (*n* = 12), and bilateral CP (*n* = 5) (21, 23, 24, 28–32, 35, 37–41). Several studies also reported motor impairment severity using the Manual Ability Classification System (MACS) and Gross Motor Function Classification System (GMFCS), with distributions summarized in Table 2.

**Table 2:** Overview of population demographics in included studies.

**Sample size**	**Age (mean, SD)**	**Gender**	**CP subtype**	**GMFCS levels**	**MACS levels**
Total participants: (*n* = 293) Children with CP: (*n* = 213)	10.81 ± 3.69 years (*n* = 123)^*^	169 males 116 females	Hemiplegic (*n* = 64) Spastic (*n* = 95) Dyskinetic (*n* = 12) Bilateral (*n* = 5)	I (*n* = 6) II (*n* = 2) III (*n* = 5) IV (*n* = 4)	I (*n* = 18) II (*n* = 17) III (*n* = 15) IV (*n* = 6)

CP, cerebral palsy; GMFCS, Gross Motor Functional Classification System; MACS, Manual Ability Classification System; SD, standard deviation.

^*^The mean age and standard deviation calculation excluded three studies (21, 23, 33) that only reported the age range or mean age of participants.

### 3.3 Neurotechnology characteristics

The included studies investigated a wide variety of wearable neurotechnology systems designed to facilitate upper extremity rehabilitation in children with CP (Table 3). Despite their variety, several common characteristics and thematic groupings emerged based on design features, control mechanisms, wearability, and modes of stimulation. These categories included: (i) surface EMG-triggered stimulation systems (38.1%), (ii) game-based or virtual reality (VR) platforms with integrated haptic feedback or electrical stimulation (23.8%), (iii) robot-assisted devices incorporating haptic feedback or stimulation (19.05%), and (iv) wearable garments embedded with electrical or vibrotactile stimulators (19.05%).

**Table 3:** Overview of neurotechnology characteristics in the included studies.

**Author**	**Neurotechnology description**	**Neurotechnology logistics**	**Availability and cost**
Arkkukangas et al. (38)	Device: Electro-Dress Mollii^®^ Wearability: Full-body suit Neurotechnology features: Embedded electrodes providing transcutaneous electrical stimulation to the antagonists of the selected spastic muscles, customly designed for each child. Control unit: Detachable control unit sends electrical signals and activates electrodes placed on individually selected antagonist muscles. Intended use: Designed to reduce spasticity and improve motor function through reciprocal inhibition among children with CP.	Sponsored research: Study not sponsored Device manufacturer: Inerventions AB, a Swedish MedTech company (47). FDA/CE approval: No	Availability: Available at select clinics and rehabilitation facilities in Europe. Price range: £5,800 to purchase, £196 for fitting assessment, £540 per month to rent + £2,000 deposit.
Azzam (21)	Device: Mesh Glove Sensory Stimulation Wearability: Full-hand mesh glove with two rubber electrodes placed on the dorsal and ventral forearm. Neurotechnology Features: Delivers NMES via dual-channel stimulation (30–40 Hz; 300 μs pulse duration) to create a tickling sensation without visible or palpable contraction at threshold sensory stimulation; some children started at 20 Hz. Control unit: Fixed pulse duration; frequency adjustable based on tolerance. Intended use: Reduces hypertonia, enhances voluntary movement and hand awareness, and decreases spatial hemi-neglect.	Sponsored research: No Device manufacturer: Information not provided FDA/CE approval: Information not provided	Availability: Information not provided Price range: Information not provided
Bloom et al. (24)	Device: Portable Silent Surface EMG Unit Wearability: Three-part system (electrode head, ground strap, belt pack) worn on the affected upper extremity. Neurotechnology features: Provides surface EMG biofeedback from muscle bellies to support motor activation in children with negative motor signs. Control unit: Simple on/off switch. Intended use: Improve UE function based on areas listed in the Goal Attainment Scale: movement-based (e.g., wrist extension, grip strength) and function-based (e.g., hold a spoon for 1 min., use affected hand as an assist hand)	Sponsored research: No Device manufacturer: David Profitt FDA/CE approval: No	Availability: Prototype Price range: N/A
Bortone et al. (22, 26)	Device: Immersive VR Neuro-Rehabilitation System with Wearable Haptics Wearability: Lightweight, dual-finger (thumb and index) wearable haptic devices designed to minimize interference with hand movement and task performance. Neurotechnology features: Combines head-mounted display (HMD), optical tracking, and wearable haptic devices delivering 3-DoF low-frequency fingertip deformation. Provides visual, auditory, and haptic feedback to enhance sensory input. Control unit: Actuated by electromagnetic RC servo motors, controlled via a Teensy 3.2 board and Pololu Wixel wireless transceiver. Fully wireless and battery-powered to avoid movement constraints. Intended use: Promotes engagement and multisensory feedback during motor tasks; VR environment allows customized, adaptive rehab exercises.	Sponsored research: This work was partially supported by the “Wearable Haptics for robot and humans” (short: WEARHAP) project Device manufacturer: HMD: Oculus Rift DK2, optical infrared tracking system (Optitrack V120) (48). FDA/CE approval: No	Availability: Prototype Price range: N/A
Butzer et al. (29) and Lieber et al. (25)	Device: Pediatric Hand Exoskeleton (PEXO) Wearability: Fully wearable hand exoskeleton; available in two sizes—kidPEXO (ages 6–9) and juvenilePEXO (ages 10–12). Attaches via finger and wrist straps (leaves palm exposed for sensory input) or a Velcro glove (easier to don but covers palm). Neurotechnology features: Uses a three-layer spring system for hand movement. Controlled via an EMG sensor and custom interface (includes comparator, LED, and threshold slider). The device activates when the EMG signal exceeds a set threshold. The EMG control unit can be placed on any superficial muscle to record the EMG signal and thereby control PEXO. Control unit: Exoskeleton toggles between open/close via any TTL trigger through an audio jack in the backpack (e.g., Buddy Buttons or EMG unit), customizable to user or therapist preference (large diameter pushbuttons). Intended use: Supports task-based training by assisting grasp during functional activities in clinical or home settings.	Sponsored research: The study was funded by the ETH Foundation Device manufacturer: ETH Zurich (49), Rehabilitation Engineering Laboratory FDA/CE approval: No	Availability: Prototype Price range: N/A
Casellato et al. (30) and Lunardini et al. (40)	Device: EMG-Based Vibrotactile Biofeedback Device Wearability: A two-part system with electrode head and belt pack is worn on the affected upper extremity. Neurotechnology Features: Electrode detects EMG activity from the target muscle and provides sensory augmentation through a vibration motor that is proportional to the magnitude of the EMG. Control unit: Uses Bayesian estimation to calculate EMG amplitude and control a silent vibration motor. Intended use: Enhances motor learning and control by increasing sensory feedback and awareness of muscle activity	Sponsored research: No Device manufacturer: 3D motion tracking system: OEPSystem, BTS; EMG system: FreeEMG 300, BTS Bioengineering, Milan, Italy (50). FDA/CE approval: CE Mark	Availability: This device can be purchased from research suppliers, such as BIOPAC Price range: N/A
Constantino et al. (39)	Device: Custom Myoelectric Elbow-Wrist-Hand Orthosis (MEWHO) Wearability: Surface EMG sensors integrated into the upper cuff (biceps/triceps) and distal forearm (wrist/finger flexors and extensors) of the orthosis. Neurotechnology features: Detects EMG signals during muscle contraction to trigger joint movement. Control unit: The EMG signals activate motors positioned on the elbow and distal forearm that move the joints in the desired direction to help augment weak muscles in a paretic limb. Intended use: Supports performance of daily activities across clinic, home, and community settings.	Sponsored research: No Device manufacturer: Myoelectric Orthosis MyoPro (51) (Myomo Inc., Cambridge, MA, USA). FDA/CE approval: FDA-approved through the 510(k) Premarket Notification process and is classified as a Class II device.	Availability: The device can be purchased and is available for use at therapy centers. Price range: The Motion W and G devices are $33,480.90 and $65,871.74, respectively. The devices may be covered by CMS.
Fluet et al. (31, 32)	Device: New Jersey Institute of Technology Robot Assisted Virtual Rehabilitation (NJIT-RAVR) Wearability: Seated setup with foot support; the affected upper extremity is stabilized in a positional splint. Neurotechnology features: A 6-DoF admittance-controlled robotic arm (Haptic Master with ring gimbal) uses 3D force, position, and velocity sensing to generate reactive motion and simulate haptic effects (e.g., springs, dampers, global forces). Control unit: Measures user-generated force to drive real-time motion in a customizable virtual environment with haptic feedback. Intended use: Enhances motor function in children with proximal UE impairments through 3D movement training of the shoulder, elbow, and forearm.	Sponsored research: No Device manufacturer: NJIT-RAVR was developed by researchers at NJIT, but the HapticMaster component was developed by Moog FCS Robotics (52). FDA/CE approval: No	Availability: NJIT Price range: N/A
Fu et al. (34)	Device: Custom video game with Contralaterally Controlled Functional Electrical Stimulation (CCFES) Wearability: Fingerless mitt with thumb/finger openings for ease of use; bend sensor on index finger secured with 3D-printed rings. Three transcutaneous electrodes are placed over the extensor muscles of all four fingers and the thumb to fully open the more-affected hand of each participant. Neurotechnology features: Bend sensor on less affected hand triggers FES to extensor muscles (via 3 surface electrodes) on the more affected hand to assist with hand opening. Control unit: Custom battery-powered stimulator from the Cleveland FES Center; movement of the less affected hand controls stimulation via the game interface. The CCFES is stimulated by opening the less affected hand while wearing a command glove. Intended use: Delivers task-specific hand therapy by engaging weak muscles in functional, goal-directed movements through interactive gaming.	Sponsored research: Yes, MetroHealth Medical Center Device manufacturer: Video game design: Unity game development engine (Unity Technologies, San Francisco, CA); running on Windows 8 (Microsoft Corp., Redmond, WA). Bend sensor (Images SI, Inc., Staten Island, NY) on the more-affected hand. Sensor- attached to a fingerless mitt (53) (Handana, Austin, TX). FDA/CE approval: No	Availability: Prototype Price range: N/A
Gerber et al. (27)	Device: Portable YouGrabber System Wearability: Neoprene glove (available in 4 sizes) with silicone finger rings (6 sizes); sensor boxes attach via Velcro. Neurotechnology features: Sensor “boxes” contain magnetometers, accelerometers, and bending sensors to track hand/finger movement. Overhead camera monitors spatial position; vibration units on the back of the hand provide haptic feedback. Control unit: Computer-based training system calibrates ROM, adapts tasks to individual needs, and provides performance feedback. Intended use: Enhances neuroplasticity and upper limb function (e.g., grasp, shoulder, elbow, wrist, ROM) through game-based training with vibration feedback.	Sponsored research: No Device manufacturer: YouRehab, a Swiss company specializing in rehab tech FDA/CE approval: Not available	Availability: Available at various hospitals and rehabilitation centers in the US; however, one may contact YouRehab to see where exactly it is distributed Price range: Not available
Hernandez et al. (35)	Device: Falcon Haptic Feedback Therapy Gaming System Wearability: Custom grips for ADL-relevant hand positions; forearm mount isolates wrist extension; sling supports arm weight. Neurotechnology features: Includes Novint Falcon, 3 interchangeable grips, baseplate for wrist rotation, forearm mount, dominant-hand trigger button, arm sling, and therapist-controlled software for customizing haptic feedback and ROM. Games are designed for bimanual use and therapeutic movement. Control unit: Participants use the hemiplegic hand to control movements in X/Y axes (≥3.5 cm from center); the dominant hand presses a big button to trigger game actions. Haptic feedback is tailored by therapists. Intended use: Facilitates high-repetition, feedback-rich movement practice to support motor learning and improve upper extremity function in children with CP.	Sponsored research: No Device manufacturer: The Novint Falcon (Novint Technologies, Albuquerque, New Mexico, US) (54) FDA/CE approval: No	Availability: Components of the gaming system can be purchased separately. Price range: The Falcon Haptic Controller is ~$500 USD, not including the gaming system and other adaptations.
Kuo et al. (36)	Device: Gloreha Sinfonia Wearability: This device focuses on the distal part of the upper limb and uses a dynamic support system that facilitates whole limb function. Neurotechnology features: Combines a soft, glove-like exoskeleton with a dynamic support system, which detects the movement of each finger, partially or completely supports a patient's movement, and performs task-oriented exercises through the object used. Control unit: Not specified in the article or on the company's website. Intended use: Improve independence in ADLs, hand, and motor function in individuals with motor deficits from pediatric stroke.	Sponsored research: No Device manufacturer: Indrogent, Lumezzane, Italy. BTL Robotics FDA/CE approval: CE mark	Availability: The device can be purchased from the website, and price quotes are given upon request Price range: Information not available
Kuroda et al. (37) and Shimizu et al. (28)	Device: Hybrid Assistive Limb^®^- Single Joint (HAL-SJ) + Integrated Volitional Control Electrical Stimulation (IVES). Wearability: One cuff is worn around the biceps/triceps, and the other wraps around the upper forearm. The motor is placed at the elbow. Neurotechnology features: HAL-SJ is a single-joint wearable device (elbow extension/flexion), combined with IVES, which produces intense electrical stimulation in direct proportion to the amplitude of the target muscles' voluntary monitor EMG. Control unit: HAL-SJ uses a bioelectrical signal (BES)- based control system that demonstrates joint torque assistance with the wearer's voluntary drive. Intended use: Improve upper limb function and ADL performance.	Sponsored research: No Device manufacturer: HAL: Cyberdyne Inc., Tsukuba, Japan; IVES: OG Giken, Okayama, Japan (55) FDA/CE approval: HAL-SJ has a CE marking (CE 0197) and meets the Medical Device Directive requirements (EU).	Availability: The device can be rented through the Cyberdyne website, which offers membership plans. Price range: *¥191,400* for a 3 3-month rental.
Muccio et al. (33)	Device: AxioBionics' BioSleeve NMES Device Wearability: Fabric Sleeve that covers the entire affected UE and is secured with two straps around the trunk. Can be worn underneath clothing. Neurotechnology features: The device is embedded with 6 BioGel Velcro electrodes and wiring to distribute current from the Axiobionics 4-channel neuromuscular stimulator to the three sets of muscles (deltoid, triceps, finger extensors). Control unit: Axiobionics 4-channel neuromuscular stimulator. The level of stimulation provided by this control unit was limited to an intensity that delivered the max range of motion without overextending the joint. For reference, the required levels of stimulation are labeled on the front face of the stimulator. Intended use: To determine if this device could increase arm mobility in the long term.	Sponsored research: Philip Muccio is the owner of Axiobionics. Device manufacturer: AxioBionics FDA/CE approval: Yes	Availability: Physician prescribed (56) Price range: Not available; can be covered by insurance or other funding options available.
Seo et al. (41)	Device: TheraBracelet Wearable and Smartphone App Wearability: A custom watch-like wearable device worn on the affected upper extremity. Neurotechnology features: Delivers sensory stimulation at 60% of the user's sensory threshold, determined via app-based questions. Control unit: Stimulation is triggered by an onboard accelerometer when movement of the affected limb is detected, ensuring delivery only during sensorimotor engagement. Intended use: Enhances neural activity during upper extremity sensorimotor tasks to support functional hand use and developmental progress in children with CP.	Sponsored research: The lead author holds the patent for the vibrotactile stimulation of this device. No external funding. Device manufacturer: Zucker Institute for Innovation Commercialization, Canopy Design Lab, LLC, Charleston, SC, USA, and Fount LLC, Mount Pleasant, SC, USA. FDA/CE approval: No	Availability: Gen 3 device is ready for manufacturing, although the product is not yet commercially available Price range: N/A
Sporea et al. (23)	Device: Functional Electrical Stimulation (FES) combined with Robot Assisted Therapy (RAT) Wearability: Lightweight orthosis worn on the forearm Neurotechnology features: Orthosis is embedded with five surface electrodes positioned on the forearm, sending electrical impulses to the muscles that control hand function while the patient is engaging in RAT programs, given real-time tactile and audiovisual feedback. Control unit: FES is controlled by a small, electronic device that sends low-voltage electrical signals to trigger muscle activation based on pre-set electrical current characteristics (intensity, frequency, threshold). Intended use: Combining FES and RAT serves to improve patient performance, normalize muscle tone, increase ROM and speed reaction, reduce pain, and improve coordination by stimulating nerves and muscles, imitating natural electrical brain signals.	Sponsored research: No Device manufacturer: Not available FDA/CE approval: Yes (57)	Availability: Both the RAT program and FES are readily available online and in inpatient and outpatient rehab centers. Price range: Average price for RAT is $5,152. Price of FES highly varies depending on brand, type, home-use vs. clinic use, ranging from hundreds to thousands.

ADL, activities of daily living; CMS, Centers for Medicare and Medicaid; CP, cerebral palsy; DoF, degrees of freedom; EMG, electromyography; FES, functional electrical stimulation; Hz, hertz; LED, light-emitting diode; N/A, not available; RC, radio controlled; RAT, robot-assisted therapy; NMES, neuromuscular electrical stimulation; ROM, range of motion; TTL, Transistor-Transistor Logic; UE, upper extremity; VR, virtual reality; μs, microseconds; 3D, three-dimensional.

Devices in this category leveraged surface EMG technology to detect volitional muscle activity and trigger electrical or vibrotactile stimulation. Examples included the Hybrid Assistive Limb^®^-Single Joint (HAL-SJ) system with Integrated Volitional Control Electrical Stimulation (IVES) (28, 37), the EMG-Based Vibrotactile Biofeedback Device (30, 40), the Pediatric Hand Exoskeleton (PEXO) (25, 29), a Portable Silent Surface EMG Unit (24), and a custom Myoelectric Elbow-Wrist-Hand Orthosis (MEWHO) (39). These systems often operated as closed-loop feedback mechanisms, using real-time bioelectrical signals to modulate stimulation parameters and enhance movement control. This integration of neuromuscular feedback aimed to reinforce motor learning by aligning external stimulation with voluntary effort.

Virtual reality and gamified platforms, often combined with haptic feedback and/or electrical stimulation to the upper extremity, were designed to create engaging, task-specific environments that promote motivation and repetitive practice. Systems such as the Immersive VR Neuro-Rehabilitation System with Wearable Haptics (22, 26), the YouGrabber^®^ glove-based home exergame (27), a contralaterally controlled functional electrical stimulation (CCFES) video game interface (34), and the Falcon haptic-feedback gaming system (35) enabled interactive upper limb tasks. These technology systems aimed to enhance motivation and participation by embedding therapeutic goals into immersive and enjoyable activities.

Robot-assisted devices provided guided movements, gravity compensation, and joint-specific support, often paired with haptic feedback, EMG control, or functional electrical stimulation (FES). These included the Gloreha Sinfonia (36), a dynamic hand exoskeleton; the Robot-Assisted Virtual Rehabilitation (RAVR) system with a six-degree-of-freedom robotic arm (31, 32); and a combined FES orthosis with robot-assisted therapy (RAT) (23). These systems aimed to facilitate neuroplasticity through precise, repetitive motion training while incorporating sensorimotor feedback.

Several studies evaluated garments embedded with stimulatory components to reduce spasticity and enhance neuromuscular activation. Examples included the Mollii^®^ suit, a full-body transcutaneous electrical stimulation (TENS) system tailored to individual spasticity profiles (38); AxioBionics' BioSleeve NMES Device (33); the Mesh Glove Sensory Stimulation device for localized hand stimulation (21); and the TheraBracelet, a wrist-worn vibratory stimulator linked to movement and paired with a mobile app (41). These devices prioritized ease of wear and consistent stimulation, particularly for home-based or long-duration use.

### 3.4 Neurotechnology availability and cost

Information regarding device availability and cost was limited across the included studies and was supplemented through searches of company websites and publicly available market data (Table 3). Among the wearable neurotechnology systems, only the Gloreha Sinfonia (36), Axiobionics' Biosleeve (33), and Cyberdyne's Hybrid Assistive Limb^®^ (28, 37) are currently commercially available to the public on company websites. Other devices, such as the Electro-Dress Mollii^®^ (38), EMG-Based Vibrotactile Biofeedback Device (30, 40), MEWHO (39), NJIT-RAVR (31, 32), YouGrabber (27), and FES + RAT (23), are only accessible through medical institutions, rehabilitation centers, or collaborative research programs. Several systems remained in prototype stages, including the Immersive VR Neuro-Rehabilitation System (22, 26), PEXO (25, 29), the Portable Silent Surface EMG unit (24), the CCFES-video game system (34), and the TheraBracelet (41). Price data was also limited and revealed substantial cost variation among neurotechnology systems (Table 3). Only five devices had obtained FDA or CE regulatory approval (23, 28, 30, 36, 37, 39, 40), highlighting the novelty of wearable neurotechnology devices and limited availability for the pediatric population.

### 3.5 Interventions and protocols

Intervention protocols varied widely across studies in terms of treatment intensity, wear schedules, and co-interventions (Supplementary material 2). Most interventions were delivered over multiple weeks, with wear times ranging from 15 min to 8 h per day, and frequencies of 1–7 sessions per week. In contrast, some feasibility and pilot studies occurred over a single session (25, 26, 29).

Three studies explored the use of wearable devices exclusively in home or community settings (24, 27, 41). Five of the studies' protocols included both clinical interventions and home-based programs (21, 33, 34, 37, 39), while the remaining studies utilized the devices solely during therapist-led sessions conducted in hospital or outpatient clinic settings. All studies, except those focused exclusively on home or community use, included therapist-guided upper extremity rehabilitation with wearable neurotechnology devices. Although specific intervention protocols differed across studies, many incorporated conventional upper extremity rehabilitation strategies, including graduated exercises, facilitation and inhibition techniques, constraint-induced movement therapy, bimanual training, grasp and reach tasks, and guided functional task practice. Key characteristics of each study's interventions are summarized in Supplementary material 2.

### 3.6 Study outcomes

Feasibility and usability were assessed in many studies through measures such as device tolerance, time worn, ease of donning, and participant satisfaction via logs or interviews. Feasibility was generally positive across studies, with high adherence rates and minimal adverse events reported (24–29, 36–39, 41). However, usability challenges were noted, including device bulk, fatigue, need for therapist assistance, and sensory discomfort from device weight, warmth, or skin irritation in some cases. Additionally, a few studies reported technical issues with neurotechnology systems, particularly at-home systems, such as the Portable YouGrabber System (27), TheraBracelet (41), and the Portable Silent Surface EMG unit (24). Social and psychological barriers also emerged, with some participants expressing reluctance to wear devices in school or public settings due to self-consciousness or fear of damaging the equipment, even when perceiving physical benefits (39, 41).

A broad range of outcome measures were used to assess motor and functional performance, including standardized tools such as the Assisting Hand Assessment (AHA), Box and Blocks Test (BBT), ABILHAND-kids, Action Research Arm Test (ARAT), Modified Ashworth Scale (MAS), and Functional Independence Measure (FIM), as well as EMG-based muscle activity analysis. Many studies reported improvements in motor outcomes, including grip strength, hand use, range of motion, grasp and release, and muscular recruitment. However, heterogeneity in study designs and outcome measures limited direct comparisons. Only a subset of studies used standardized assessments, and long-term follow-up data were largely unavailable.

## 4 Discussion

This scoping review identified 21 studies investigating 16 wearable neurotechnology systems designed to support upper extremity rehabilitation in children with CP. Despite considerable heterogeneity in device design, study methodologies, intervention protocols, and outcome measures, the findings indicate that many of these technologies are feasible, well-tolerated, and demonstrate promising potential for improving motor function when integrated with task-specific, repetitive training.

Across studies, wearable neurotechnology devices were associated with improvements in various domains of upper extremity function, including grip strength, range of motion, muscle activation, coordination, and bimanual task performance. Systems that incorporated gamification, virtual reality, or home-based integration frequently reported enhanced engagement and usability, which are critical factors for adherence and functional carryover in pediatric rehabilitation (42). Additionally, several wearable systems employed closed-loop control mechanisms, such as EMG-triggered stimulation or movement-activated sensory feedback. These technologies closely align with established principles of motor learning and neuroplasticity, which emphasize active engagement, self-initiation of movement, and real-time feedback (10). Such features are believed to facilitate cortical reorganization and enhance functional recovery (8, 43, 44). Notably, devices such as BioSleeve, HAL-SJ, and MEWHO orthosis exemplify systems that support task-specific training with potential applications in daily routines (28, 33, 37, 39). Their design reflects a shift toward portable, personalized neurorehabilitation strategies that accommodate real-world use (11, 12).

Beyond device function, implementation context emerged as a notable consideration. Home-based systems offer the possibility of distributed, intensive practice, addressing limitations in access to pediatric therapy and intensive programs (11, 14). Many studies revealed the importance of motivation, autonomy, and adaptability, especially for children navigating social and environmental challenges. This echoes findings from previous neurorehabilitation research, where motivation and autonomy, often enhanced through biofeedback and gamified systems, were key drivers of positive outcomes (42).

While the adult neurorehabilitation literature has extensively documented the benefits of robotic and sensor-based technologies for stroke and spinal cord injury, studies targeting children with CP remain sparse (13, 15, 16). This review adds to the limited but growing body of work emphasizing wearable, non-invasive neurotechnology specifically tailored to the pediatric population. These devices stand out for their affordability, portability, and scalability, positioning them well for home and community integration, a priority in pediatric rehabilitation models (12).

Nevertheless, implementation challenges remain. Many devices are early-stage prototypes, lacking regulatory approval and formal clinical guidelines. Few studies offered detailed implementation guidelines, creating uncertainty for therapists and families regarding setup, customization, safety, and integration into existing care models (9, 10). These findings are consistent with previous calls for more standardized outcome measures and more rigorous study designs in pediatric neurotechnology research (10, 45, 46). Practical concerns, including device bulkiness, donning difficulty, and sensory discomfort, were common. Social stigma associated with wearing conspicuous technology in public settings also emerged as a barrier, underscoring the need for discreet, child-friendly designs that balance therapeutic benefits with usability.

### 4.1 Strengths and limitations

A strength of this review is its comprehensive scope and inclusion of diverse study designs, technologies, and intervention approaches, supported by a broad search strategy across multiple databases and gray literature sources. The focus on children with CP fills an important gap in the literature, as most prior reviews have focused on adult populations or non-wearable technologies.

However, the current evidence base remains preliminary, with most studies comprising small sample sizes and low levels of evidence, such as feasibility studies and case series. Only three randomized controlled trials were identified, only one study included long-term follow-up, and no studies included direct measures of cortical change. Many studies involved co-interventions, making it difficult to isolate the effects of the neurotechnology alone. Furthermore, intervention protocols varied widely across studies in terms of duration, intensity, frequency, and task specificity, which may introduce bias and limit the comparability and generalizability of reported outcomes. Variability in outcome measures further restricted cross-study comparability. As with all scoping reviews, no formal quality appraisal or meta-analysis was conducted, and results should be interpreted accordingly.

### 4.2 Future directions

Future research should follow a structured roadmap to advance the clinical translation of wearable neurotechnology for upper extremity rehabilitation in children with cerebral palsy. The first priority is the systematic validation of emerging devices through regulatory pathways to ensure safety, usability, and efficacy. Depending on device readiness, this process may range from benchmark and safety testing to full-scale regulatory approval (e.g., FDA or CE certification). Large-scale efficacy trials are then warranted to evaluate device performance across varied rehabilitation contexts, including home-based and community settings. Establishing an international working group to identify standardized and clinically meaningful outcome measures, encompassing functional performance, participation, and patient-reported experiences, will be essential to enhance data comparability and enable meta-analytic synthesis.

Subsequent implementation research should focus on integrating wearable systems into routine pediatric rehabilitation practice while addressing barriers to clinician and family adoption. Studies should examine training requirements, customization needs, and interdisciplinary coordination to optimize usability and adherence. Incorporating neurophysiological and neuroimaging assessments (e.g., electroencephalogram (EEG) and functional magnetic resonance imaging (fMRI) within these studies could provide mechanistic insight into neuroplastic changes associated with device use. Finally, long-term follow-up and real-world effectiveness studies are needed to evaluate sustained outcomes, user satisfaction, and scalability. Economic analyses addressing cost-effectiveness, accessibility, and reimbursement potential will be critical to inform sustainable integration into pediatric rehabilitation services. Collectively, these steps represent a progressive trajectory, from regulatory validation to implementation and long-term impact, that will guide the responsible advancement of wearable neurotechnology toward widespread clinical adoption.

## 5 Conclusion

This scoping review provides an overview of the current landscape of wearable, non-invasive neurotechnology systems used to support upper extremity rehabilitation in children with CP. The findings demonstrate an increasing interest in leveraging wearable neurotechnology devices, such as EMG-triggered stimulators, virtual reality systems, robotic interfaces, and sensor-embedded garments that provide sensory feedback or electrical stimulation, to enhance motor function, promote neuroplasticity, and increase access to high-frequency, task-specific practice. While the studies reviewed suggest wearable neurotechnology devices are generally feasible, well-tolerated, and promising in improving motor outcomes, the literature remains early in development, with limited high-level evidence and inconsistent outcome reporting. Wearable neurotechnology offers an exciting frontier in pediatric neurorehabilitation. With continued development, collaborative design, and translational research, these systems may play a critical role in supporting functional independence and improving quality of life for children with CP.

## Data Availability

The original contributions presented in the study are included in the article/Supplementary material, further inquiries can be directed to the corresponding author.
