# Supplementary material for: Wearable neurotechnology systems for upper extremity rehabilitation in children with cerebral palsy: a scoping review
Source: Front Neurol. 2025 Nov 5;16:1663596. doi: 10.3389/fneur.2025.1663596 (PMC12635914; doi:10.3389/fneur.2025.1663596)
Supplement: Supplementary file 1 [file Table_1.docx]

**Ovid (Medline): Search ran on 4/15/25 and produced 776 results.**

- Cerebral Palsy/ or ("cerebral palsy" or "little* disease*" or "spastic diplegia*" or "perinatal stroke" or "prenatal stroke*" or "neonatal stroke*" or "infant stroke" or "pediatric stroke" or spasticit* or hemipares* or "perinatal brain injur*" or "prenatal brain injur*" or "neonatal brain injur*").mp.
- Transcranial Direct Current Stimulation/ or ("peripheral magnetic stimulation*" or "transcranial direct current stimulation*" or "non-invasive brain stimulation").mp.
- exp Electric Stimulation Therapy/ or ("electric* stimulat*" or neuromodulat*).mp.
- exp Electromyography/ or (electromyograph* or electromyogram*).mp.
- exp Neurological Rehabilitation/ or ("neurologic* rehabilitation*" or neurorehabilitation*).mp.
- exp Wearable Electronic Devices/ or ("wearable electronic device*" or "wearable device*" or "wearable technolog*" or "electronic skin" or "wearable computer*" or neuroprosthetic* or neuro-prosthetic*).mp.
- exp Occupational Therapy/ or ("occupational therap*" or ergotherap*).mp.
- 2 or 3 or 4 or 5 or 6 or 7
- exp Child/ or exp Adolescent/ or exp Infant/ or exp Pediatrics/ or (pediatric* or paediatric* or child* or kid* or toddler* or infan* or bab* or neonat* or newborn* or new-born* or adolescent* or teen* or juvenil*).mp.
- exp Upper Extremity/ or ("upper extremit*" or "upper limb*" or "membrum superius" or hand* or arm* or shoulder* or wrist* or elbow*).mp.
- 1 and 8 and 9 and 10

**CINAHL: Search ran on 4/15/25 and produced 522 results.**

- (MH "Cerebral Palsy") OR ("cerebral palsy" OR "little* disease*" OR "spastic diplegia*" OR "perinatal stroke" OR "prenatal stroke*" OR "neonatal stroke*" OR "infant stroke" OR "pediatric stroke" OR spasticit* OR hemipares* OR "perinatal brain injur*" OR "prenatal brain injur*" OR "neonatal brain injur*")
- (MH "Transcranial Direct Current Stimulation") OR ("peripheral magnetic stimulation*" OR "transcranial direct current stimulation*" OR "non-invasive brain stimulation")
- (MH "Electric Stimulation+") OR ("electric* stimulat*" OR neuromodulat*)
- (MH "Electromyography") OR (electromyograph* OR electromyogram*)
- (MH "Wearable Sensors+") OR ("wearable electronic device*" OR "wearable device*" OR "wearable technolog*" OR "electronic skin" OR "wearable computer*" OR neuroprosthetic* OR neuro-prosthetic*)
- (MH "Occupational Therapy+") OR ("occupational therap*" OR ergotherap*)
- ("neurologic* rehabilitation*" OR neurorehabilitation*)
- S2 OR S3 OR S4 OR S5 OR S6 OR S7
- (MH "Child+") OR (MH "Infant+") OR (MH "Pediatrics+") OR (pediatric* OR paediatric* OR child* OR kid* OR toddler* OR infan* OR bab* OR neonat* OR newborn* OR new-born* OR adolescent* OR teen* OR juvenil*)
- (MH "Upper Extremity+") OR ("upper extremit*" OR "upper limb*" OR "membrum superius" OR hand* OR arm* OR shoulder* OR wrist* OR elbow*)
- S1 AND S8 AND S9 AND S10

**Scopus: Search ran on 4/15/25 and produced 11,308 results.**

( ( TITLE-ABS-KEY ( "peripheral magnetic stimulation*" OR "transcranial direct current stimulation*" OR "non-invasive brain stimulation" ) OR TITLE-ABS-KEY ( "electric* stimulat*" OR neuromodulat* ) OR TITLE-ABS-KEY ( electromyograph* OR electromyogram* ) OR TITLE-ABS-KEY ( "neurologic* rehabilitation*" OR neurorehabilitation* ) OR TITLE-ABS-KEY ( "wearable electronic device*" OR "wearable device*" OR "wearable technolog*" OR "electronic skin" OR "wearable computer*" OR neuroprosthetic* OR neuro-prosthetic* ) OR TITLE-ABS-KEY ( "occupational therap*" OR ergotherap* ) ) AND TITLE-ABS-KEY ( "cerebral palsy" OR "little* disease*" OR "spastic diplegia*" OR "perinatal stroke" OR "prenatal stroke*" OR "neonatal stroke*" OR "infant stroke" OR "pediatric stroke" OR spasticit* OR hemipares* OR "perinatal brain injur*" OR "prenatal brain injur*" OR "neonatal brain injur*" ) AND TITLE-ABS-KEY ( pediatric* OR paediatric* OR child* OR kid* OR toddler* OR infan* OR bab* OR neonat* OR newborn* OR new-born* OR adolescent* OR teen* OR juvenil* ) AND TITLE-ABS-KEY ( "upper extremit*" OR "upper limb*" OR "membrum superius" OR hand* OR arm* OR shoulder* OR wrist* OR elbow* ) )

**PsycInfo: Search ran on 4/15/25 and produced 283 results.**

- exp Cerebral Palsy/ or ("cerebral palsy" or "little* disease*" or "spastic diplegia*" or "perinatal stroke" or "prenatal stroke*" or "neonatal stroke*" or "infant stroke" or "pediatric stroke" or spasticit* or hemipares* or "perinatal brain injur*" or "prenatal brain injur*" or "neonatal brain injur*").mp.
- Transcranial Direct Current Stimulation/ or ("peripheral magnetic stimulation*" or "transcranial direct current stimulation*" or "non-invasive brain stimulation").mp.
- exp Noninvasive Brain Stimulation/ or exp Electrical Brain Stimulation/ or exp Neuromodulation/ or ("electric* stimulat*" or neuromodulat*).mp.
- exp Electromyography/ or (electromyograph* or electromyogram*).mp.
- exp Neurorehabilitation/ or ("neurologic* rehabilitation*" or neurorehabilitation*).mp.
- exp Wearable Devices/ or ("wearable electronic device*" or "wearable device*" or "wearable technolog*" or "electronic skin" or "wearable computer*" or neuroprosthetic* or neuro-prosthetic*).mp.
- exp Occupational Therapy/ or ("occupational therap*" or ergotherap*).mp.
- 2 or 3 or 4 or 5 or 6 or 7
- exp Pediatrics/ or (pediatric* or paediatric* or child* or kid* or toddler* or infan* or bab* or neonat* or newborn* or new-born* or adolescent* or teen* or juvenil*).mp.
- exp "Arm (Anatomy)"/ or exp "Hand (Anatomy)"/ or ("upper extremit*" or "upper limb*" or "membrum superius" or hand* or arm* or shoulder* or wrist* or elbow*).mp.
- 1 and 8 and 9 and 10
